# Restoration of metabolic inflammation-related ghrelin resistance by weight loss

**DOI:** 10.1530/JME-17-0192

**Published:** 2017-12-12

**Authors:** Farhana Naznin, Koji Toshinai, T M Zaved Waise, Tadashi Okada, Hideyuki Sakoda, Masamitsu Nakazato

**Affiliations:** 1 Division of NeurologyRespirology, Endocrinology and Metabolism, Department of Internal Medicine, Faculty of Medicine, University of Miyazaki, Miyazaki, Japan; 2 Department of Sports and FitnessFaculty of Wellness, Shigakkan University, Obu, Japan; 3 Agency for Medical Research and Development-Core Research for Evolutional Medical Science and Technology (AMED-CREST)Japan Agency for Medical Research and Development, Tokyo, Japan

**Keywords:** ghrelin, inflammation, high-fat diet, vagal nerve, hypothalamus

## Abstract

High-fat diet (HFD)-induced metabolic inflammation in the central and peripheral organs contributes to the pathogenesis of obesity. Long-term HFD blunts signaling by ghrelin, a gastric-derived orexigenic peptide, in the vagal afferent nerve via a mechanism involving *in situ* activation of inflammation. This study was undertaken to investigate whether ghrelin resistance is associated with progressive development of metabolic inflammation. In mice, ghrelin’s orexigenic activity was abolished 2–4 weeks after the commencement of HFD (60% of energy from fat), consistent with the timing of accumulation and activation of macrophages and microglia in the nodose ganglion and hypothalamus. Calorie-restricted weight loss after 12-week HFD feeding restored ghrelin responsiveness and alleviated the upregulation of macrophage/microglia activation markers and inflammatory cytokines. HSP72, a chaperone protein, was upregulated in the hypothalamus of HFD-fed mice, potentially contributing to prevention of irreversible neuron damage. These results demonstrate that ghrelin resistance is reversible following reversal of the HFD-induced inflammation and obesity phenotypes.

## Introduction

Obesity is a long-term disturbance of energy metabolism in which energy intake exceeds energy expenditure over a prolonged period (Moehlecke *et al*. 2016). The control of food intake and body weight involves central nervous system integration of information from the peripheral nervous system and humoral signals from the gastrointestinal tract (Morton *et al*. 2006, Begg & Woods 2013). Vagal afferents that innervate digestive organs transmit sensory information from their endings to the nucleus of the tractus solitarius (NTS) in the medulla oblongata, terminating in distinct hypothalamic nuclei involved in feeding and energy homeostasis. The vagus afferent nerve is a pseudounipolar neuron whose cell body is located in the nodose ganglion, making one projection to the NTS and the other to peripheral organs. Vagal afferent neurons express receptors for gut peptides such as ghrelin, cholecystokinin and glucagon-like peptide 1 that regulate feeding and energy homeostasis (Zarbin *et al*. 1981, Burdyga *et al*. 2006, Cummings & Overduin 2007).

Ghrelin, a peptide primarily produced in the stomach, stimulates feeding (Tschöp *et al*. 2000, Nakazato *et al*. 2001). The growth hormone secretagogue receptor (GHSR), also known as the ghrelin receptor, is synthesized in vagal afferent neurons and transported to the stomach by axonal transport (Date *et al*. 2002). Electrophysiological studies indicate that ghrelin hyperpolarizes nodose ganglion neurons by activating K_ATP_ conductance (Grabauskas *et al*. 2015), thereby attenuating electrical activity of the vagal afferent (Date *et al*. 2002). This ghrelin signal is sent to the NTS and relayed via the noradrenergic pathway to hypothalamic neurons expressing the orexigenic neuropeptides agouti-related peptide (AgRP) and neuropeptide Y (NPY) (Date *et al*. 2006). GHSR, a G-protein-coupled receptor, activates the intracellular Ca^2+^ signaling and AMP-activated protein kinase (AMPK) signaling pathways (Kohno *et al*. 2003, Hardie 2004). Previous studies showed that neither central nor peripheral ghrelin administration induced feeding in diet-induced obese (DIO) mice fed a high-fat diet (HFD, 60% of energy from fat) for 12 or 16 weeks (Perreault *et al*. 2004, Briggs *et al*. 2010, Gardiner *et al*. 2010, Naznin *et al*. 2015). Another study demonstrated that ghrelin resistance developed 3 weeks after the start of HFD feeding (in that case, 23.5% energy from fat) (Briggs *et al*. 2014). In addition, we showed that ghrelin’s effects on energy expenditure, suppression of vagus afferent electrical activity and neuronal activation in the hypothalamic arcuate nucleus were abolished in 12-week HFD-fed mice (Naznin* et al*. 2015). All of these DIO experiments used C57BL/6J mice, which develop severe obesity, hyperglycemia and insulin resistance (Reed *et al*. 2007). Consumption of HFD induces immune cell-mediated tissue inflammation in the gut, adipose tissue, liver, skeletal muscle and hypothalamus (De Souza *et al*. 2005, Schenk *et al*. 2008, Thaler *et al*. 2012, Hotamisligil 2017), which causes macrophage-associated pathological alterations and insulin resistance. We confirmed that HFD caused accumulation and activation of macrophages and microglia in the nodose ganglion and hypothalamus of mice (Naznin* et al*. 2015). These inflammatory responses resulted in the reduction of *Ghsr* expression in these neuronal tissues, causing impaired transmission of gastric-derived ghrelin signals to the hypothalamus (Naznin* et al*. 2015). Ghrelin resistance under HFD is thought to be caused by dysregulation of ghrelin signaling via the vagal afferent. Ghrelin resistance is the consequence of several factors in addition to dysregulation of the ghrelin signaling via vagal nerve. Peripheral ghrelin reaches the hypothalamus by passive diffusion through the fenestrated capillaries of the median eminence, which project to the ventromedial part of the ARC and ultimately target NPY/AgRP neurons to induce a metabolic response (Schaeffer *et al*. 2013).

In this study, we sought to determine when ghrelin resistance develops during HFD exposure of mice and to investigate whether the onset of ghrelin resistance is linked to inflammation in the nodose ganglion and hypothalamus. Subsequently, we investigated whether calorie-restricted weight loss could reverse inflammation in these neuronal tissues in order to restore ghrelin sensitivity.

## Materials and methods

### Animals and dietary protocols

Male C57BL/6J mice (6-week-old male, 20–21 g, Charles River Laboratories) were maintained in individual cages under controlled temperature (21–23°C) and light (light on: 08:00–20:00) conditions. All animal experiments were approved by the Animal Care and Use Committee of University of Miyazaki.

### Study 1: time course of ghrelin resistance

**Figure 1:**
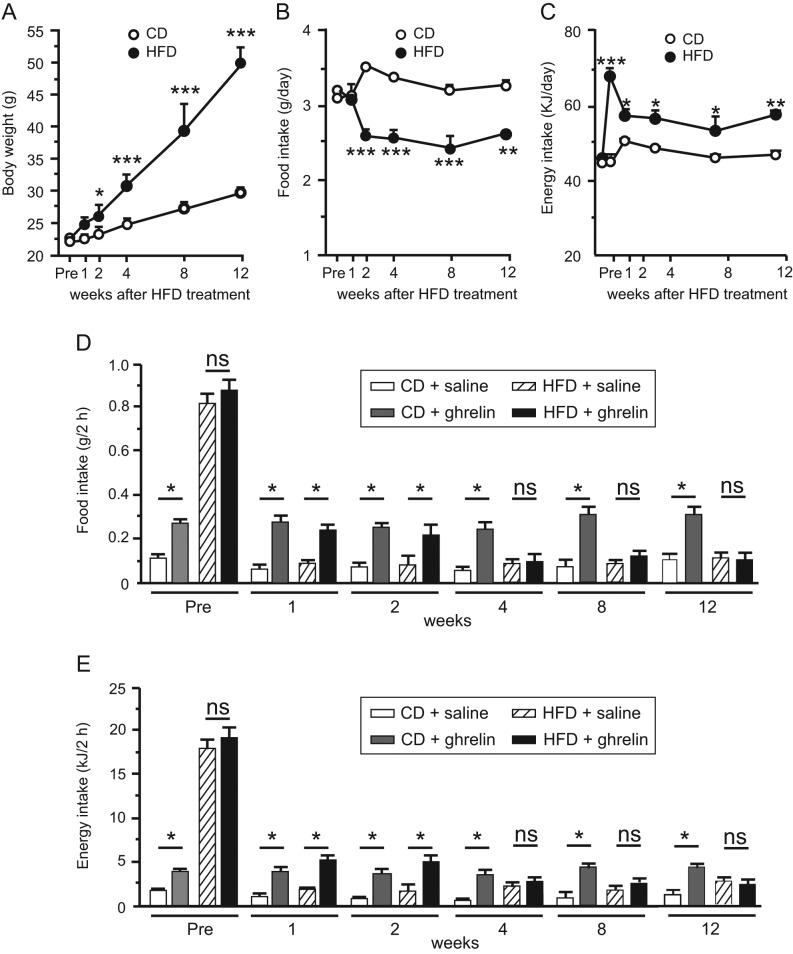
Changes in body weight (A), food intake (B) and energy intake (C) of mice fed CD or HFD for 12 weeks. Effects of HFD on ghrelin-induced food intake (D) and energy intake (E) in 1-, 2-, 4-, 8-, or 12-week CD- or HFD-fed mice. Values are means ± s.e.m. *n* = 6–8. *P < 0.05, **P < 0.01, ***P < 0.001.

Mice were maintained on either chow diet (CD) (12.3% fat, 59.2% carbohydrate, 28.5% protein, 14.2 kJ/g; CLEA Rodent Diet CE-2, CLEA Japan, Tokyo, Japan) or HFD (60% fat, 20% carbohydrate, 20% protein, 21.9 kJ/g; no. D12492; Research Diets, New Brunswick, NJ, USA), with free access to food, for 12 weeks. Respective percentages of saturated fatty acid, monounsaturated fatty acid and polyunsaturated fatty acid in the HFD were 32, 36 and 32%. Body weight and 24-h food intake were measured weekly. ‘Pre’ indicates the period before starting the HFD (Fig. 1).

### Study 2: caloric restriction

Mice were assigned to one of three diet groups: HFD for 16 weeks (HFD group); HFD for 12 weeks followed by a switch to CD for 2 weeks and then calorie restriction (60% *ad libitum*) for 2 weeks until their body weights reached those of age-matched CD-fed controls (HFD-CD/CR group); and standard laboratory chow for 16 weeks (CD group). .

### Food intake experiments

Mice fed CD or HFD (*n* = 6 per group) were transferred to single cages and maintained for 1 week. They were acclimatized by subcutaneous (s.c.) injections of saline once daily for 3 days before the feeding experiment. Ghrelin (60 nmol/kg BW; Peptide Institute, Osaka, Japan) was administered subcutaneously (s.c.), and 2-h food intake was measured.

### Energy expenditure

Mice fed CD or HFD (*n* = 4–12 per group) were housed in a metabolic chamber (Shinfactory, Fukuoka, Japan) for 1 week. They received s.c. injection of ghrelin (60 nmol/kg BW) or saline at 10:00 and were then returned to the chamber. Energy expenditure was measured for 2 h in an Oxymax (Columbus Instruments, Columbus, OH, USA). Mice were deprived of food during the measurement.

### Measurement of blood parameters

Mice were fasted from 09:00 to 14:00 before blood collection by tail prick. Blood glucose was measured using a glucometer (Terumo, Tokyo, Japan), and plasma insulin was measured using a mouse insulin EIA kit (Morinaga Institute of Biological Science, Yokohama, Japan). For plasma ghrelin measurements, mice were anesthetized deeply with sodium pentobarbital (Abbott Laboratories), and blood samples were collected by cardiac puncture. Plasma ghrelin was measured using the active ghrelin ELISA Kit (Mitsubishi Chemical Medience, Tokyo, Japan; intra- and inter-assay precision coefficient of variation <10%, assay range 2.5–160 fmol/mL). All samples were measured in duplicate.

### Real-time polymerase chain reaction (RT-PCR)

The nodose ganglion and hypothalamus were quickly removed from anesthetized CD- or HFD-fed mice. The tissue was stored in RNAlater (Life Technologies) at −80°C. For RNA extraction, samples were placed in tubes containing autoclaved glass beads (425–600 µm) (Sigma-Aldrich) and vortexed for 6 min on a TissueLyser (Qiagen). Total RNA was extracted using the RiboPure kit (Ambion). RT-PCR was conducted on a Thermal Cycler Dice Real-Time System II (Takara Bio) using SYBR Premix Ex Taq (2×) (Takara Bio). Primer sets for RT-PCR are shown in Table 1. mRNA levels for each gene were normalized against the level of *Tbp* mRNA in the same sample, used as an internal control.

**Table 1:** Primer sequences for genes used in the real-time PCR analysis.

Gene	Forward primer	Reverse primer
*Ghsr*	ATCACCTCTGGGTCTTGTTGCTG	GCTGAATGGCTCATTGTAGTCCTG
*Tlr4*	GGAAGTTCACATAGCTGAATGAC	CAAGGCATGTCCAGAAATGAGA
*Iba1*	AGCTGCCTGTCTTAACCTGCATC	TTCTGGGACCGTTCTCACACTTC
*Il6*	CCACTTCACAAGTCGGAGGCTTA	CCAGTTTGGTAGCATCCATCATTTC
*Tnfα*	TATGGCCCAGACCCTCACA	GGAGTAGACAAGGTACAACCCATC
*Ap2a*	ACCCTCGTGTGGAGCCTAAGA	AGGTTCACAAACGCGACAGA
*Bhlhe22*	CTGACAATTGGCAAGTGATGAAAG	CTCCTGGCTCAGAATCAAGATG
*Nrebp*	TGACAGTGGCCCTGACCATC	GGTACAAGGCCCATTGCTTGA
*Tbp*	CATTCTCAAACTCTGACCACTGCAC	CAGCCAAGATTCACGGTAGATACAA

### Immunohistochemistry

Nodose ganglia and whole brains (*n* = 4−5 per group) were immersed in 4% paraformaldehyde/phosphate buffer for 24 h at 4°C, incubated for 24 h in PB containing 20% sucrose, quickly frozen on dry ice and cut into 8-µm slices using a cryostat at −20°C. Sections were blocked for 5 min in protein-block serum-free solution (Dako), and then incubated overnight at 4°C with rabbit anti-Iba1 (1:10,000; Wako Pure Chemicals), rat anti-CD86 (1:100; Abcam), mouse anti-HSP72 (1:50; Enzo Life Sciences, New York, NY, USA) or mouse anti-NeuN (1:200; Millipore, Chemicon International). Immunofluorescence was performed with Alexa Fluor 488-labeled anti-rabbit secondary antibody or Alexa Fluor 594-labeled anti-mouse secondary antibody (both 1:400; Invitrogen). Images were captured on an OLYMPUS AX-7 fluorescence microscope (Olympus). Cells immunoreactive for Iba1, CD86 or HSP72 in the nodose ganglion and hypothalamus of three mice were counted manually in three to five sections per mouse using the cellSens imaging software (Olympus). Quantitation was performed in a blinded fashion.

### Statistical analysis

Statistical analyses were performed by one- or two-way ANOVA followed by a Bonferroni’s post-test for multiple comparisons, as appropriate. When two mean values were compared, analysis was performed by Mann–Whitney, Wilcoxon or unpaired *t*-test. All data are expressed as means ± s.e.m. *P* < 0.05 was considered to be statistically significant.

## Results

### Effects of HFD on body weight, food intake and ghrelin responses

**Figure 2:**
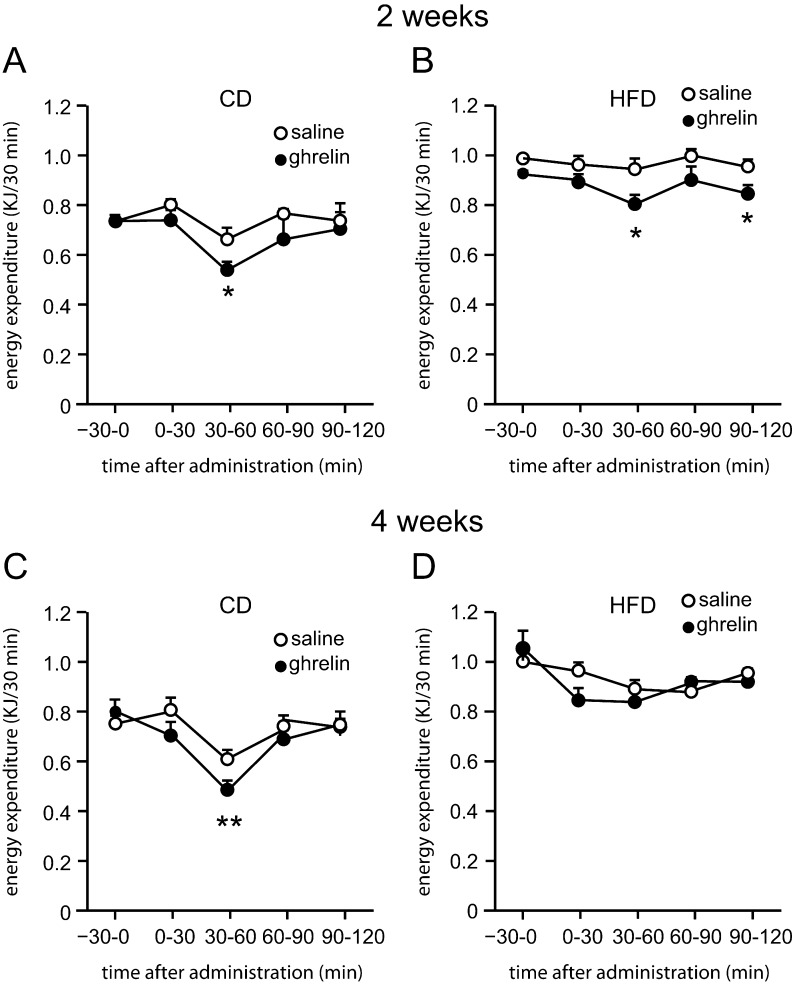
Effect of ghrelin on energy expenditure in DIO mice. Energy expenditure in 2-week CD- (A) or HFD-fed mice (B), and 4-week CD- (C) or HFD-fed mice (D) receiving ghrelin. Values are means ± s.e.m. *n* = 4–12. *P < 0.05, **P < 0.01, ***P < 0.001.

Body weights of mice on HFD were significantly higher than those of CD-fed mice 2 weeks after the initiation of HFD feeding (Fig. 1A). Average food intake of HFD-fed mice was lower than that of CD-fed mice from 2 to 12 weeks (Fig. 1B), although the energy intake of HFD-fed mice was greater during this period (Fig. 1C). Ghrelin administration significantly increased food intake in both 1- and 2-week HFD-fed mice, but not in 4-, 8- or 12-week HFD-fed mice (Fig. 1D and E). Ghrelin administration decreased energy expenditure in both 2- and 4-week CD-fed mice and 2-week HFD-fed mice, but not in 4-week HFD-fed mice (Fig. 2A, B, C and D).

### mRNA expression of *Ghsr*, inflammatory genes and transcriptional factors

**Figure 3:**
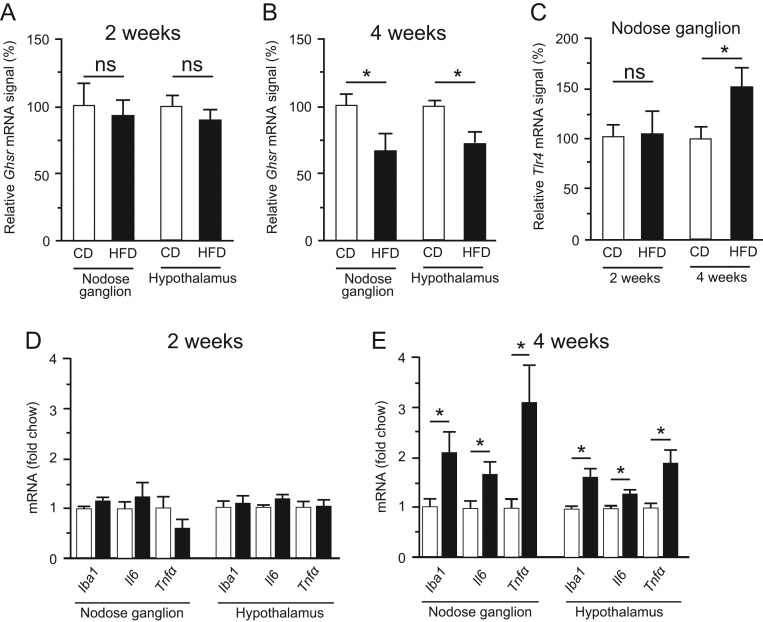
*Ghsr* mRNA expression in the nodose ganglion (A) and hypothalamus (B) of mice fed CD or HFD for 2 (A) or 4 (B) weeks. mRNA levels of *Tlr4* (C) in the nodose ganglion of 2- or 4-week CD- or HFD-fed mice. mRNA levels of *Iba1*, *Il-6*, and* Tnfα* in the nodose ganglion and hypothalamus of 2- (D) or 4- (E) week CD- or HFD-fed mice. mRNA levels were normalized against the level of *Tbp* mRNA (a housekeeping gene) in the same sample, and the normalized values are presented as fold change relative to CD. Values are means ± s.e.m. *n* = 8–10. *P < 0.05 vs CD.

**Figure 4:**
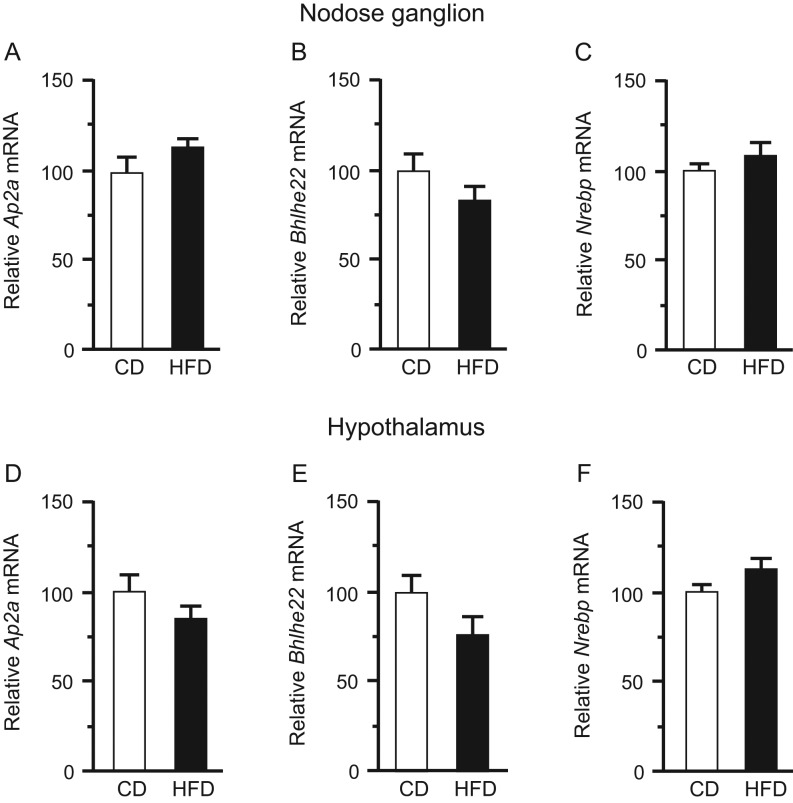
mRNA levels of *Ap2a*, *Bhlhe22*, and* Nrebp* in the nodose ganglion (A, B and C) and hypothalamus (D, E and F) of CD- or HFD-fed mice at 12 weeks. mRNA levels were normalized against the level of *Tbp* mRNA in the same sample. *n* = 9–14. Values are means ± s.e.m.

*Ghsr* mRNA levels in both the nodose ganglion and hypothalamus did not differ significantly between CD-fed and HFD-fed mice at 2 weeks, but at 4 weeks were significantly lower in HFD-fed mice (Fig. 3A and B). *Tlr4* expression in the nodose ganglion did not differ significantly between CD- and HFD-fed mice at 2 weeks, but was significantly higher in HFD-fed mice at 4 weeks (Fig. 3C). Two-week HFD feeding did not modulate the expression of any of the genes investigated in the nodose ganglion or hypothalamus, in comparison with 2-week CD feeding, whereas 4-week HFD feeding significantly upregulated the *Iba1*,* Il6* and *Tnfα* mRNAs in comparison with 4-week CD feeding (Fig. 3D and E). Expression of* Ap2a*,* Bhlhe22* and* Nrebp*, which encode transcription factors of *Ghsr*, in the nodose ganglion and hypothalamus did not differ significantly between the CD and HFD groups at 12 weeks (Fig. 4).

### Effect of HFD on immunohistochemical markers of inflammation

**Figure 5:**
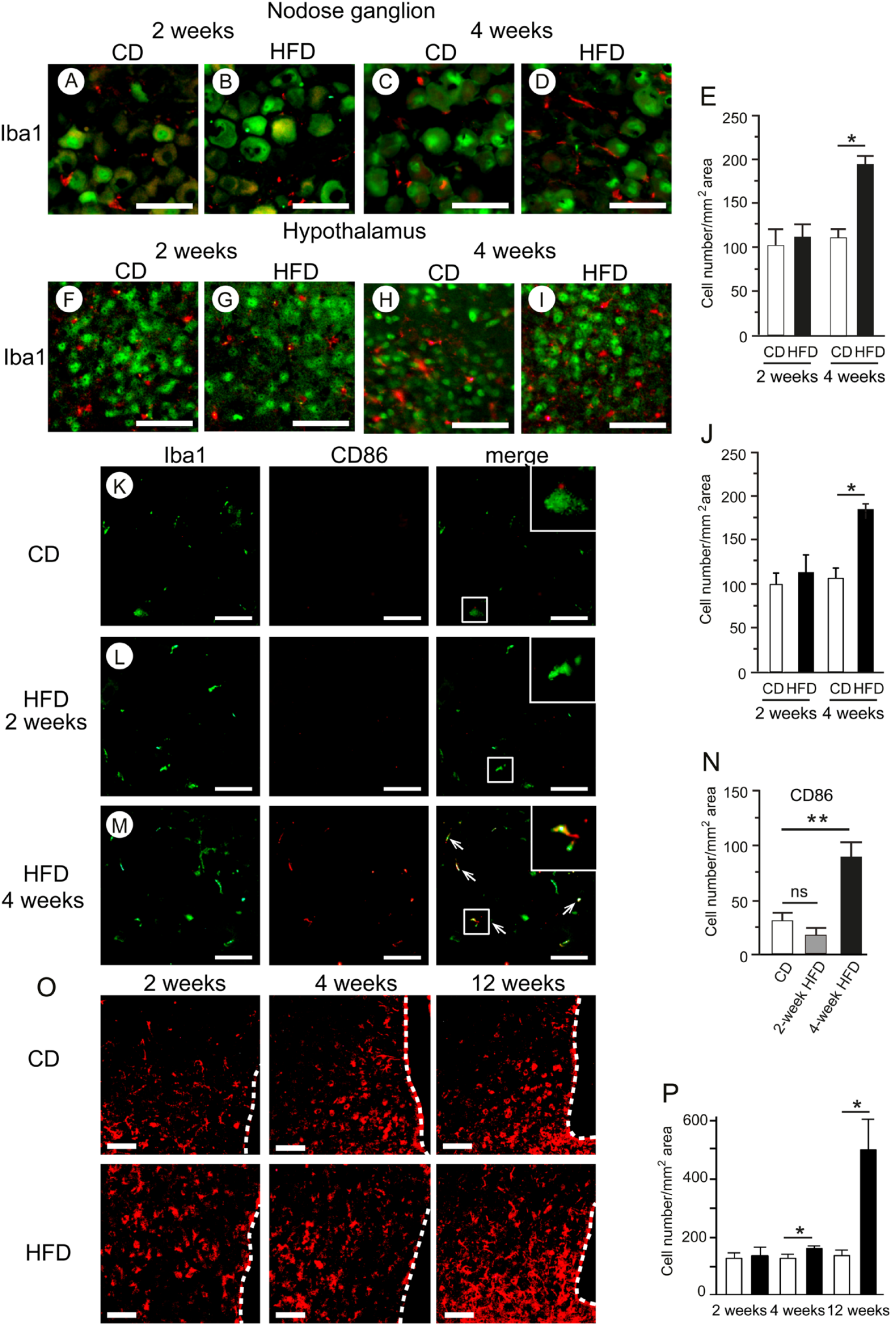
HFD-induced macrophage accumulation in the nodose ganglion and hypothalamus. Representative immunohistochemical detections of Iba1 (red) and NeuN (green) in the nodose ganglion (A, B, C and D) and hypothalamus (F, G, H and I) of 2- or 4-week CD- or HFD-fed mice. Numbers of cells stained with anti-Iba1 antibody in the nodose ganglion (E) and hypothalamus (J). Representative images of Iba1 (green) and CD86 (red) in the nodose ganglion (K, L and M) of 2- or 4-week CD- or HFD-fed mice. Arrows indicate co-localization of CD86 with Iba1. Numbers of CD86-positive cells in the nodose ganglion (N). Histochemical analyses of HSP72 in the hypothalamus of 2-, 4- or 12-week HFD-fed mice (O). Numbers of HSP72-positive cells in the hypothalamus (P). Values are means ± s.e.m. *n* = 4–5. *P < 0.05, **P < 0.01 vs CD. Scale bars, 50 μm. Dotted lines indicate the third ventricle.

The average number of macrophages stained with anti-Iba1 antibody in the nodose ganglion (Fig. 5A, B, C, D and E) and hypothalamus (Fig. 5F, G, H, I and J) was significantly higher in HFD-fed mice than that in CD-fed mice at 4 weeks, whereas no difference was observed between these two groups at 2 weeks. The expression level of the M1 macrophage marker CD86 in the nodose ganglion was significantly higher in HFD-fed mice than in CD-fed mice at 4 weeks, but no difference between groups was apparent at 2 weeks (Fig. 5K, L, M and N). Macrophages immunoreactive for CD86 in the HFD group were larger and were morphologically rounded and more ramified, than those in the CD group (Fig. 5M). The average number of HSP72-positive cells in the hypothalamus was significantly greater in the HFD group than that in the CD group after 4 weeks of HFD feeding (Fig. 5O and P).

### Effects of caloric restriction on ghrelin sensitivity and inflammation

**Figure 6:**
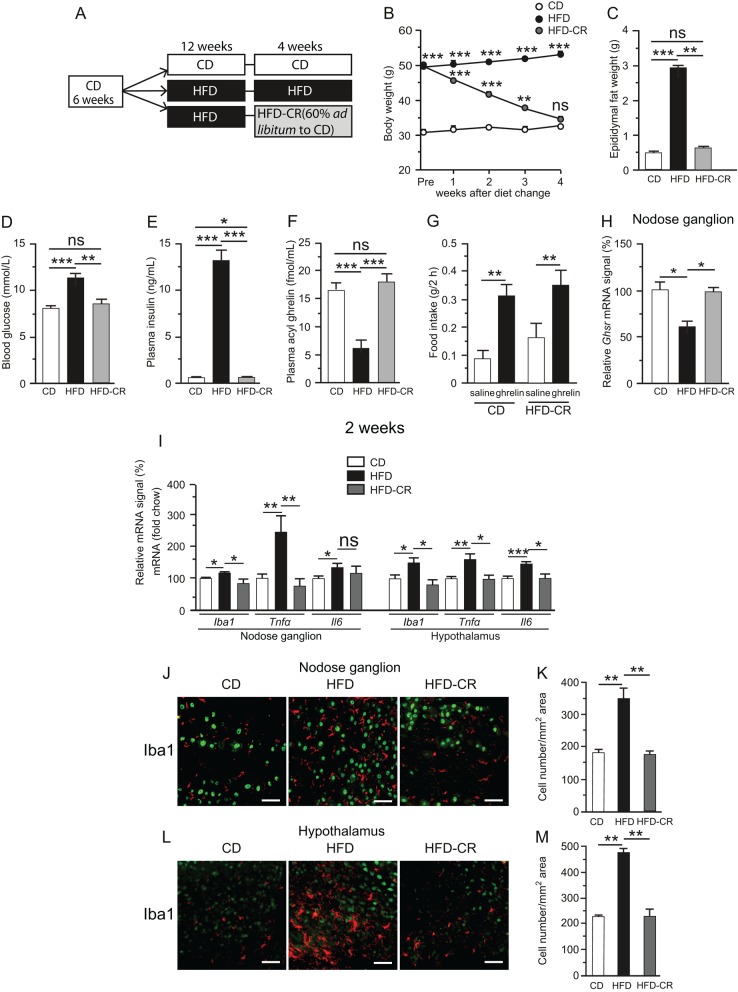
Schematic representation of caloric restriction experiment (A). Mice were initially placed on 12-week CD followed by 4 additional weeks of chow (CD), 12-week HFD followed by 4 additional weeks of HFD (HFD), or 12-week HFD followed by switching to chow and caloric restriction to 60% of *ad libitum* intake (HFD-CR). Body weight, epididymal fat weight, blood glucose, plasma insulin, and ghrelin levels of CD, HFD and HFD-CR mice (B, C, D, E and F). **P < 0.01, ***P < 0.001 vs CD in (B). *n* = 6–8. Effect of diet change on ghrelin-induced food intake (G). mRNA expression of *Ghsr* (H) in the nodose ganglion. mRNA levels of *Iba1*,* Tnfα*, and* Il-6* in the nodose ganglion and hypothalamus of CD, HFD, or HFD-CR mice (I). *n* = 8–10. Immunohistochemical detections of Iba1 (J), and numbers of cells stained with Iba1 antibody in the nodose ganglion in CD, HFD, and HFD-CR mice (K). *n* = 4. Values are means ± s.e.m. Iba1, red; NeuN, green. *P < 0.05, **P < 0.01, ***P < 0.001 in (C) to (M). Scale bars, 50 μm.

The difference in body weight between the CD and HFD-CD/CR groups became insignificant at the end of 4-week caloric restriction (Fig. 6B). In the HFD-CD/CR group, epididymal fat weight, fasting blood glucose and plasma insulin and ghrelin returned to normal levels (Fig. 6C, D, E and F). Ghrelin administration significantly stimulated food intake in the HFD-CD/CR group (Fig. 6G). The *Ghsr* mRNA level in the nodose ganglion was significantly lower in 16-week HFD mice than that in CD-fed mice, whereas the level in the HFD-CR group did not differ from that in the CD group (Fig. 6H). *Iba1*,* Il6* and *Tnfα* mRNAs in both the nodose ganglion and hypothalamus of the HFD-CD/CR group were not upregulated relative to the CD group (Fig. 6I). The average number of Iba1-positive macrophages in the nodose ganglion and hypothalamus was also significantly higher in the HFD group than that in both the CD and HFD-CD/CR groups, whereas no significant difference was observed between the CD and HFD-CD/CR groups (Fig. 6J, K, L and M).

## Discussion

We investigated whether exposure to HFD could affect ghrelin responsiveness in mice. Metabolic inflammation in both the nodose ganglion and hypothalamic arcuate nucleus developed 2–4 weeks after the start of the HFD. The anabolic activities were also abolished during this interval. In a previous study, we found that 1-day HFD feeding in C57BL/6J mice induced inflammatory responses, concomitant with upregulation of activated macrophage/microglia markers and inflammatory cytokines, in the nodose ganglia and arcuate nuclei (Waise *et al*. 2015). Thaler and coworkers found that the HFD-induced inflammatory response in the rat hypothalamus exhibited a complex ‘on–off–on’ pattern in which the initial inflammatory response developed 3 days after the start of HFD (60% energy from fat), followed by a decline to baseline from days 7 to 14, and a subsequent return to elevated levels by day 28 (Thaler *et al*. 2012). This hypothalamic inflammatory response occurred rapidly, even before substantial weight gain, in response to HFD feeding. Microglia/macrophages play regulatory roles in the innate immune system of the nervous system by responding to pathological insults. Acute inflammatory cytokine production, which develops rapidly in response to HFD in animals, is crucial for the initiation of adaptive immune responses, and allows the host to adapt to pathological conditions (Thaler *et al*. 2012, Waise *et al*. 2015). Chronic inflammatory signals in the nodose ganglion and hypothalamus cause microglia/macrophages to assume a ramified morphology. In response to homeostatic perturbations, these more active cells produce cytokines, reactive oxygen species and other toxic mediators (Neumann *et al*. 2006, Hanani 2010). The time course of inflammatory responses observed in this study was well correlated with the development of ghrelin resistance at 4 weeks. Iba1, ionized calcium-binding adaptor molecule 1, is a marker of microglia/macrophage activation in the nervous system (Ito *et al*. 1998). We found that 4-week HFD-induced activation of microglia/macrophages and inflammatory responses in the nodose ganglion and hypothalamus, as reflected by greater numbers of both Iba1- and CD86-positive macrophage/microglia. These cells were morphologically rounded and more ramified, indicating that more activated subtypes of macrophages/microglia were present after 4 weeks of HFD. Lending support to this argument, expression of TLR4, a putative mediator of saturated fatty acid-induced inflammatory signaling, was elevated in the nodose ganglion after 4 weeks of HFD.

Berkseth and coworkers showed that 4-week CD (12% energy from fat) following 16-week HFD (60% energy from fat) in C57BL/6J mice reversed the activation of astrocytes and microglial accumulation in the hypothalamic arcuate nucleus (Berkseth *et al*. 2014). Another group demonstrated that calorie-restricted weight loss restored the ghrelin responsiveness of NPY/AgRP neurons (Briggs *et al*. 2013). In this study, the metabolic inflammation of HFD-fed mice was reversible when their body weight, fat mass and plasma glucose and insulin returned to normal levels after 4-week caloric restriction. Concomitantly, the orexigenic activity of ghrelin was restored, and plasma ghrelin and *Ghsr* expression in the nodose ganglion were also normalized. *Ghsr* expression is regulated by transcription factors including AP2, bHLH and NREBP, as well as hormones such as growth hormone, β-estradiol, triiodothyronine and hydrocortisone (Yin *et al*. 2014). mRNA levels of *Ap2a*, *Bhlhe22* and *Nrebp* in both the nodose ganglion and hypothalamus did not differ significantly between CD- and HFD-fed mice. It remains possible that unknown transcription factors of *Ghsr* are downregulated in nodose ganglion and hypothalamus of HFD mice, and the molecular mechanism by which *Ghsr* expression is reduced under HFD should be investigated in future research. Harvey and coworkers recently showed that the ghrelin/GOAT system regulates HFD-induced inflammation in the spleen and thymus (Harvey *et al*. 2017). In this study, we obtained no evidence of a direct interaction between ghrelin resistance and inflammation. However, the simultaneous development of ghrelin resistance and metabolic inflammation suggests a causal relationship between the two.

HSP72, a molecular chaperone, is expressed in multiple cell types, including neurons and glial cells. It protects stressed neurons from protein aggregation and apoptosis under various pathophysiological conditions (Foster & Brown 1997, Waise *et al*. 2015). HSP72 downregulates expression of TNFα, JNK and IKK, thereby alleviating metabolic dysregulation in obesity-induced insulin resistance (Henstridge *et al*. 2014). In this study, upregulation of HSP72 in the hypothalamus of HFD-fed mice may have helped to prevent irreversible neuron damage, allowing recovery of hypothalamic inflammation by caloric restriction.

This HFD-CD/CR experiment had some limitations. For calorie restriction study 12 weeks HFD switched to 60% *ad libitum* of the CD because their body weight did not decrease enough to match with CD-fed controls. And as these mice 60% *ad libitum* of the CD, there might be possible they eat this small amount of diet at once and be starved for long time during CR. This long-term starvation may change their metabolic status.

In conclusion, our data showed that inflammation and unresponsiveness to peripherally administered ghrelin occurred by 4 weeks of HFD feeding in C57BL/6J mice. This ghrelin resistance was reversible following reversal of the HFD-induced inflammation and obesity phenotype by caloric restriction. These findings suggest that diet-induced inflammation begets a state of ghrelin resistance and that downregulation of ghrelin biosynthesis and ghrelin resistance under HFD could prevent humans and animals from overeating. Ghrelin plays multifaceted roles in the regulation of growth hormone secretion, cell proliferation and differentiation, neuroprotection, mood, immunity and learning and memory (Muller *et al*. 2015). Future studies should investigate how disruption of ghrelin signaling in obesity influences multiple types of homeostatic regulation within the body.

### Declaration of interest

The authors declare that there is no conflict of interest that could be perceived as prejudicing the impartiality of the research reported.

### Funding

This work was supported in part by JSPS KAKENHI (No. 25293216, No. 16H05333) and A-MED CREST (No. JP17gm0610016) to M N.

### Author contribution statement

F N, Z W, and T O performed the experiments; F N, H S and M N edited the manuscript; F N, H S and M N approved the final version of manuscript; K T and M N conceived and designed the research; F N analyzed the data; F N, T O, H S and M N interpreted the experimental results; F N and T O prepared the figures and F N and M N drafted the manuscript.
